# Effectiveness of “Moro” Blood Orange *Citrus sinensis* Osbeck (Rutaceae) Standardized Extract on Weight Loss in Overweight but Otherwise Healthy Men and Women—A Randomized Double-Blind Placebo-Controlled Study

**DOI:** 10.3390/nu14030427

**Published:** 2022-01-18

**Authors:** David Briskey, Giuseppe Antonio Malfa, Amanda Rao

**Affiliations:** 1 School of Human Movement and Nutrition Sciences, University of Queensland, Brisbane, QLD 4072, Australia; d.briskey@uq.edu.au; 2 RDC Clinical, Newstead, Brisbane, QLD 4005, Australia; amanda@rdcglobal.com.au; 3 Department of Drug and Health Science, University of Catania, Viale A. Doria 6, 95125 Catania, Italy

**Keywords:** cyanidin 3-glucoside, flavonoids, BMI, fat distribution, body mass, physical activity

## Abstract

This study aimed to assess the efficacy of a blood orange *Citrus sinensis* standardized extract from “Moro” cultivar, on weight loss in overweight but otherwise healthy individuals. Anthocyanins and particularly cyanidin 3-glucoside, found in a large variety of fruits including Sicilian blood oranges, can help to counteract weight gain and to reduce body fat accumulation through the modulation of antioxidant, anti-inflammatory and metabolic pathways. In this randomized, double blind, placebo-controlled study, all participants (overweight adults aged 20–65 years old) were randomized to receive either Moro blood orange standardized extract or a placebo daily for 6-months. The primary outcome measure was change in body mass and body composition at the end of the study. After 6-months, body mass (4.2% vs. 2.2%, *p* = 0.015), body mass index (*p* = 0.019), hip (3.4 cm vs. 2.0 cm, *p* = 0.049) and waist (3.9 cm vs. 1.7 cm, *p* = 0.017) circumferences, fat mass (*p* = 0.012) and fat distribution (visceral and subcutaneous fat *p* = 0.018 and 0.006, respectively) were all significantly better in the extract supplemented group compared to the placebo (*p* < 0.05). In addition, all safety markers of liver toxicity were within the normal range throughout the study for both analyzed groups. Concluding, the present study demonstrates that Moro blood orange standardized extract may be a safe and effective option for helping with weight loss when used in conjunction with diet and exercise.

## 1. Introduction

Obesity and overweight represent a major global health and societal concern. Worldwide, the prevalence of obesity has nearly tripled since 1975 with 39% of adults over 18 years of age, overweight and 13% obese [1]. It has been widely demonstrated that many plant extracts exhibit weight loss properties and gives many health benefits due to the presence of various polyphenolic compounds [2,3,4]. Although further research into the use and effectiveness of such dietary supplements for the prevention of obesity is required [5]. A body of literature data reported that anthocyanins, polyphenolic plant secondary metabolites, polyhydroxy and polymethoxy derivatives of flavylium salts [6], suppress lipid accumulation in adipocytes trough the modulation of transcription factors regulating lipogenesis [7]. Studies by Tsuda and colleagues revealed the treatment of human adipocytes with anthocyanins resulted in the modulation of gene expression of adipocytokines, suggesting anthocyanins may play a role in regulating adipocyte function [8,9]. Therefore, recent studies demonstrated the anti-obesity effect and the healthy properties of many polyphenolic compounds including anthocyanins present in citrus fruit [10,11,12,13,14]. The “Moro” orange cultivar *Citrus sinensis* Osbeck (Rutaceae) is native to Sicily and cultivated only in a restricted area at the feet of the south side of Etna volcano (Protected Geographical Indication, PGI), it is the most highly pigmented and with the higher amount of anthocyanins among red oranges varieties [15,16,17], with deep red violet flesh ripening from December to February. In blood o range cultivars, the production of anthocyanins in the flesh and epicarp during fruit ripening is not due to particular agronomic techniques other than those common to all citrus fruits but is strictly dependent on the climate conditions and particularly by strong day-night thermal clines. The particular district of Sicily (PGI), where blood oranges are cultivated is characterized by limited rainfall and not extremely cold winter but with a temperature variation between day and night of more than 10 °C, conditions not found in any other orange-producing countries [18,19]. Moro orange is a rich source of active compounds such as hydroxycinnamic acid, flavone glycosides and ascorbic acids, including anthocyanins with an average content of about 140 mg/L [18,19,20,21]. Evidence to date has shown that blood oranges demonstrate potent antioxidant activity and cytoprotective effects that reflect their substantial role in preventing chronic pathological conditions such as cardiovascular diseases and in many forms of cancers [21,22,23,24,25]. Moreover, a recent study showed that a red orange standardized extract is able to inhibit 3T3-L1 differentiation, by downregulating adipogenic gene and enzymes together with the modulation of adiponectin secretion and leptin release [26]. Red orange intake (especially Moro juice) has also been found to limit body weight gain, enhance insulin sensitivity, and decrease serum triglycerides and total cholesterol in mice [27,28]. Similarly, a study by Titta and colleagues (2009) showed anthocyanin rich Moro juice inhibited fat accumulation in obesity induced mice despite an increase in overall energy intake [28]. Salamone and colleagues also studied the effect of Moro juice on liver steatosis in mice with diet-induced obesity over a 12-week period. Results revealed Moro juice exerted a metabolic hepatoprotective effect due to changes in the expression of several enzymes involved in lipid homeostasis [27]. Preliminary research in humans has shown 400 mg of a Moro blood orange standardized extract (Morosil^®^) supplementation in overweight yet otherwise healthy participants for 12 weeks induced a significant reduction in body weight, body mass index (BMI), waist and hip circumference in comparison to placebo group [20]. As there has only been one human trial specifically on this extract of “Moro” orange cultivar, the aim of this study is to further assess the effect of the above-mentioned standardized extract at the same dose on weight loss and on all safety markers of liver toxicity in healthy adult males and females aged between 20 and 65 years to better understand its action in conjunction with a calorie-controlled diet and exercise over a 6-month period. It was hypothesized that the supplementation with the standardized extract, in conjunction with diet and exercise, would improve weight loss in overweight but otherwise healthy male and female compared to placebo control group.

## 2. Materials and Methods

### 2.1. Clinical Trial Design, Registration and Ethical Approval

The present study was a single-site, double-blind, randomized clinical trial assessing the effectiveness of a “Moro” orange standardized extract over a 6-month supplementation period. The study was conducted by RDC Clinical (Brisbane, Australia), DXA scans were performed by Physique Science (Brisbane, Australia) and pathology analysis by Cardinal Bioresearch (Brisbane, Australia). The study utilized an active and a placebo arm to investigate weight loss in overweight, yet otherwise healthy men and women aged between 20 and 65 years. This trial was conducted in compliance with the current International Conference on Harmonization (ICH) Guideline for Good Clinical Practice (GCP), the Therapeutic Goods Administration (TGA) Note for Guidance on Good Clinical Practice, and ethical guidelines outlined in Additional Ethical Considerations. It was approved by Human Research and Ethics Committee Bellberry Limited, South Australia, Approval Number 201804237 and also registered on the Australian and New Zealand Clinical Trial Register (ANZCTR), Registration number ACTRN12618001215213. 

This study was conducted between November 2018 and June 2020 in Brisbane, Australia.

### 2.2. Trial Participants

**Figure 1:**
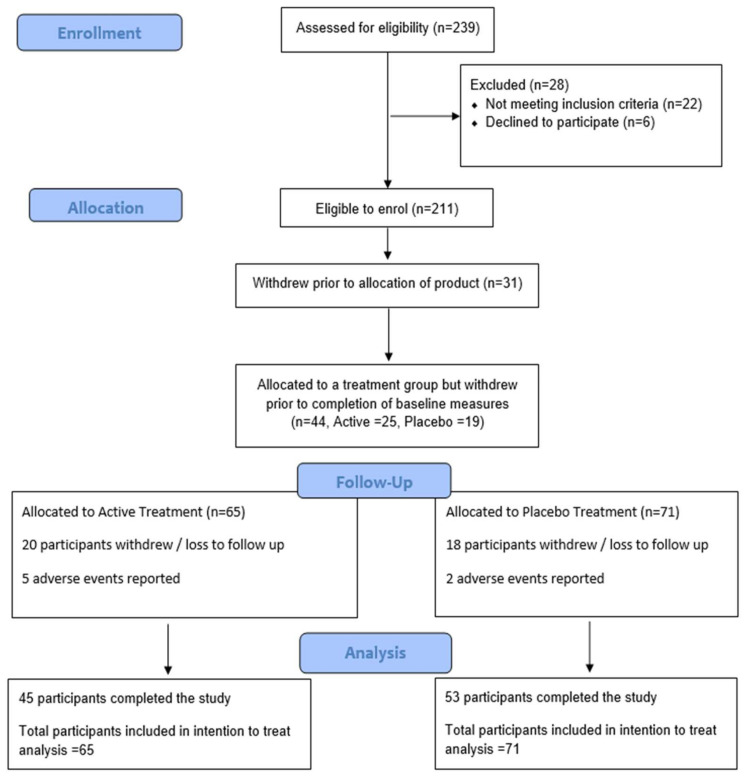
Participant Flow Diagram.

Potential participants were recruited from databases and public media outlets. Following preliminary screening via telephone, potential participants attended the clinic for an information session and provided their consent for inclusion in the trial. Consenting participants underwent a health assessment including lifestyle, current medication, medication history (history of disease and illness), recent variations in weight, alcohol and nicotine use, sleep patterns and allergies. All participants provided written informed consent prior to starting the trial. A total of 180 overweight (25 < BMI <35 kg/m^2^) yet otherwise healthy female and male volunteers aged 20 to 65 years were recruited from databases and social media from the greater Brisbane area to take part in this 6-month study (Figure 1). Participants were excluded if they had clinically significant medical conditions including, but not limited to cardiovascular, neurological, psychiatric, renal, gastrointestinal, immunological, endocrine (including uncontrolled diabetes or thyroid disease) or hematological abnormalities that were uncontrolled, significant variation in weight (more than 10%) or participation in another weight loss clinical trial in the past 3 months, current use of weight loss supplements or medications, current use of prescription medications with the exception of the oral contraceptive pill (if female), alcohol consumption of above 2 standards drinks daily, illicit drug use, smokers, malignancy or treatment for malignancy within the previous 2 years, elite or training athletes, shift workers or those with unusual sleep and/or dietary patterns, excessive caffeine intake (>4 caffeinated drinks daily). Participants were also excluded if they were found to have any allergic reactions to any of the ingredients in the active or placebo formula or had participated in any other clinical trial during the past month. Pregnant or lactating women were excluded from the study.

### 2.3. Extract Specification and Trial Product

The standardized extract (product batch number 02201901-06) called Morosil^®^ (Active) was provided by Bionap S.R.L. (Belpasso, Catania, Italy). It is obtained from the juice of “Moro” a red orange cultivar of *C. sinensis*. Medicinal name has been unified using the Kew Medicinal plant names service. The final composition of the standardized extract is as follows: cyanidin 3-O-glucoside (0.635% *w*/*w*), cyanidin 3-O-glucoside and derivatives (>0.699% *w*/*w*), hesperidin (2.13% *w*/*w*), narirutin (0.07%*w*/*w*), ferulic acid (1% *w*/*w*) and ascorbic acid (4.5% *w*/*w*), qualitative and quantitative analysis were performed using HPLC-DAD. Otherwise, UV spectroscopy quantification of total anthocyanin content was of 0.9% *w*/*w* for this analytical method. The total polyphenols content determined by HPLC is 4% (*w*/*w*), determined using calibration curves with the closest appropriate standard. Plant materials were authenticated by DNA barcoding, molecular diagnostic tests were performed by TRU-ID Ltd. (TRU-ID Ltd. Research, Guelph, ON, Canada). Certificate of authentication (Supplementary Material), detailed product specification and some parameters on purity are available on Supplementary Information. The trial product was in capsule form, each capsule containing 400 mg of standardized extract, taken with water after breakfast. This regime was selected based on current evidence for the investigational product as directed by the manufacturer and on previous clinical trials [20]. The product was stored in the trial product container at room temperature and away from direct sunlight. The placebo product for this trial was maltodextrin in a matching capsule and administered using the same procedure detailed above. Both the investigational (active) and placebo products were enclosed in containers that are identical in function and appearance.

### 2.4. Trial Description (Intervention and Study Procedures)

Once enrolled in the study, participants were randomly allocated to either the placebo comparator group (*n* = 90) or the active intervention group, “Moro” orange standardized extract (*n* = 90). Participant dietary intake, body measurements (weight, height, hip circumference, waist circumference, blood pressure and heart rate), blood parameters (alanine transaminase, aspartate aminotransferase, gamma-glutamyl transferase, total bilirubin, high density lipoproteins, low density lipoproteins, cholesterol, triglycerides, glucose, insulin, creatine, ghrelin, leptin, adiponectin) and body composition (dual-energy X-ray absorptiometry—DXA) were assessed at enrolment.

All participants received the same standard advice regarding physical activity. Specifically, participants were asked to undertake 30 min of walking 3 times per week and record all physical activity in their diaries. Participants were also asked to follow a kilojoule-controlled diet, with the daily kilojoules to be consumed by each participant calculated using the basal metabolic rate score from the individual DXA scans. Participants recorded their daily food intake in a kilojoule counter app and submit a 24-h food recall every two months during the study. Data from the diet app (Calorie Counter by FatSecret) was uploaded to FatSecret Professional software, allowing investigators to access participant diary entries. Guidance in the form of example meal plans was provided. Participants consumed a single 400 mg capsule of their allocated trial product in the morning with water after breakfast. Participants then attended the study site 1, 2, 3, 4 and 5 months after their baseline for a repeat of body measures, dietary intake and tolerance assessment. At the completion of the study (month 6), an assessment identical to that undertaken at baseline was completed. Throughout the 6-month trial, all participants were monitored for compliance with the protocol by a combination of telephone and email communications in addition to each scheduled site visit. Compliance was measured regularly during the study with participants returning the product containers at the end of the study.

### 2.5. Randomisation and Blinding

The randomization code for the products was generated by Random Allocation Software V2018, sealed Envelope, London, UK (www.sealedenvelope.com, accessed on 6 October 2020), with participants allocated to one of the two trial arms at time of enrolment. Arm 1—Active treatment, Arm 2—Placebo. All trial participants, investigators conducting the trial and the biochemist analyzing the samples were blind to which product participants were taking. The randomization code was broken upon completion of all sample analysis.

### 2.6. Outcome Measurements

The Primary outcome of weight loss and body composition included anthropometric measures of weight, body mass index (BMI), Hip and Waist circumference. Dual-energy X-ray Absorptiometry (DXA) scan measured lean muscle mass, total abdominal fat, visceral fat, subcutaneous fat, and android versus gynoid fat. The Secondary outcome measures included dietary energy intake (24-h food recall), physical activity type and frequency. Plasma samples to evaluate metabolic plasma biomarkers inclusive of fasting glucose, insulin, lipid profile and liver function were analyzed using a chemistry autoanalyzer and reagents from Biobase (Jinan, China). Plasma leptin and ghrelin were analyzed using ELISA kits from Bio-Rad (Hercules, CA, USA). Plasma adiponectin was analyzed using ELISA kits from R&D systems (Minneapolis, MN, USA). Safety and tolerability included gastrointestinal symptoms questionnaire to assess adverse reactions and gastrointestinal tolerance, and through visual analogue scale to evaluate fatigue severity (VAS-F).

### 2.7. Statistical Analysis

A sample size of 120 was required to reach statistical power (80%) based on showing a 5% difference in weight loss between groups. Due to the high drop-out rate commonly seen in weight loss studies, 180 participants were allocated to a treatment group, however 44 did not complete baseline measures. 136 participants completed baseline measurements and were included in the analysis on an intention to treat (ITT) basis where if missing data, last observation was carried forward as per ICH statistical guidelines. Statistical analysis of change from baseline between groups for each month was conducted for body composition, and anthropometric variables such as waist and hip circumference and the delta values compared between treatment groups using ANOVA, two-tailed t-tests or Mann-Whitney U tests depending on distribution of data. Dietary energy intake, physical activity history, metabolic plasma biomarkers and safety and tolerability of the product were compared at each time-point between groups using ANOVA. Results were considered statistically significant if *p* < 0.05. Gender and ethnicity data were analyzed as fixed effects, and age and height as random effects, influencing the responses to the treatments (also a fixed effect) for various outcome metrics, using fixed and mixed effects modelling. The ITT was also followed by the per protocol (PP) statistical analysis. For PP analysis, subjects who had high compliance with the protocol were judged as pass by the medical supervisor. In pp analysis, subjects were divided to active group (*n* = 41) and placebo group (*n* = 48). Sub-group participants (BMI > 25–30) were also divided to active group (*n* = 30) and placebo group (*n* = 38). The statistical significance indicates significant difference between pp-groups.

## 3. Results

Of the 180 randomized participants, 44 participants (25 active and 19 placebo) withdrew from the trial prior to completing all the baseline requirements. The most common reason being participants found the daily calorie counting too time consuming and therefore did not enter enough food diaries to meet the entry criteria. Of the 136 participants who completed the baseline requirements, 98 completed (45 active and 53 placebo) the full 6-months. Of the 38 withdrawals, there were 20 in the active treatment group, and 18 in the placebo treatment group (Figure 1). There were no significant differences between groups for exercise or dietary intake throughout the study. Both groups were well matched, with no significant differences between groups for any baseline measures (Table 1, Table 2 and Table 3).

### 3.1. Body Weight

**Figure 2:**
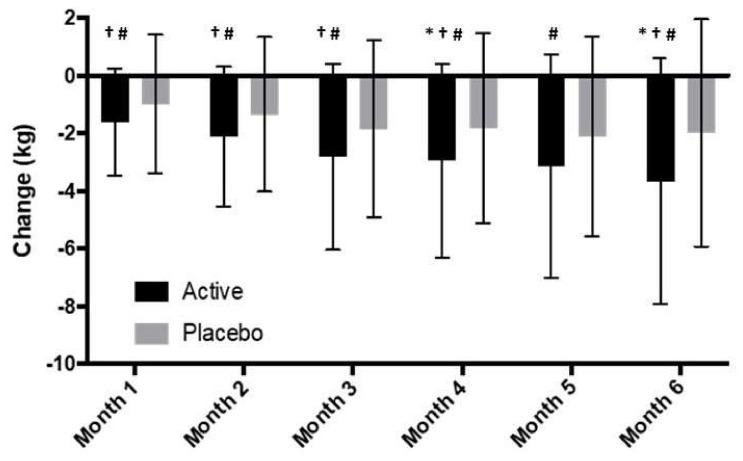
Change in body weight over 6 months for all participants; * Indicates significant difference between groups (*p* < 0.05) in ITT analysis (active *n* = 65, placebo *n* = 71). † Indicates significant difference between groups for sub-group participants (BMI > 25–30) in ITT analysis (active *n* = 48, placebo *n* = 54); # indicates significant difference between groups for sub-group participants (BMI > 25–30) in pp analysis (active *n* = 30, placebo *n* = 38).

Both groups had a significant reduction in body weight compared to baseline at the completion of the study (6-months). Participants on “Moro” orange standardized extract had a mean overall weight loss of 4.2% of starting body weight by month 6, and the placebo group had a mean overall weight loss of 2.2% of starting body weight. The change in body weight between groups was significantly different (*p* = 0.015). Additionally, 36% of the participants in the active group had a weight loss of more than 5% vs. 22.5% of the participants in the placebo group. The weight loss (kg) at the end of the study in both groups was statistically significant from baseline values, and there was a significant difference between groups at months 4 and 6 (Figure 2).

### 3.2. Hip and Waist Circumference

**Figure 3:**
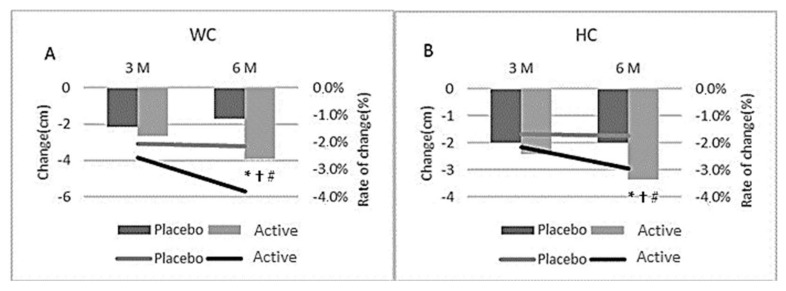
Change in waist (**A**) and hip (**B**) circumference (cm) at month 3 and 6 for all participants; * indicates significant difference between groups (*p* < 0.05) in ITT analysis (active *n* = 65, placebo *n* = 71); † indicates significant difference between groups for sub-group participants (BMI > 25–30) in ITT analysis (active *n* = 48, placebo *n* = 54); # indicates significant difference between groups for sub-group participants (BMI > 25–30) in pp analysis (active *n* = 30, placebo *n* = 38).

Both the active and placebo group had a significant reduction in waist (3.9 cm vs. 1.7 cm, respectively) and hip (3.4 cm vs. 2.0 cm, respectively) circumference from baseline following 6-month of supplementation (Table 2, Figure 3). However, the active group had a significant reduction in both hip and waist circumference at 6-months compared to the placebo group (Table 2; *p* = 0.017 and 0.049, respectively).

### 3.3. BMI

**Figure 4:**
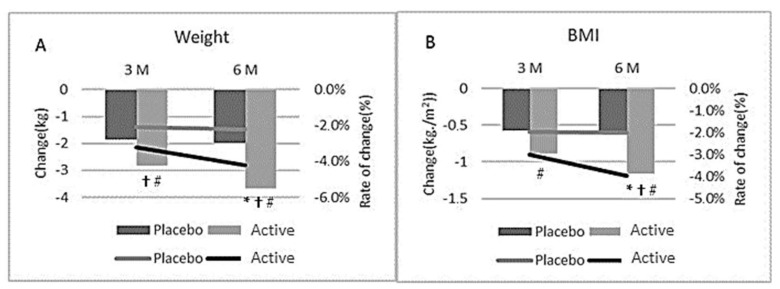
Change in Body weight (**A**) and BMI (**B**) at month 3 and 6 for all participants; * indicates significant difference between groups (*p* < 0.05) in ITT analysis (active *n* = 65, placebo *n* = 71); † indicates significant difference between groups for sub-group participants (BMI > 25–30) in ITT analysis (active *n* = 48, placebo *n* = 54); # indicates significant difference between groups for sub-group participants (BMI > 25–30) in pp analysis (active *n* = 30, placebo *n* = 38).

The average baseline BMI was 29.5 and 29.4 in the active and placebo groups, respectively (Range 25–33). Both groups had a significant reduction from baseline in BMI after 3 and 6-months of supplementation (*p* < 0.05). However, at 6-months, the active group had a significant reduction in BMI compared with the placebo group (*p* = 0.019) (Table 2 and Figure 4).

A DXA scan was conducted every 3 months during the study, if a value was missing at month 6, an intention to treat last observation carried forward imputation method was used. Of the 136 participants, 102 participants completed the DXA measurement with no significant differences between groups (*p* = 0.338). Both the active and placebo group had a significant reduction in Fat mass after 3 and 6-months of supplementation (*p* < 0.05). However, at both 3 and 6-months, the active treated group had a significant reduction in Fat mass compared with the placebo group (*p* = 0.01 and 0.012) (Table 2). This was supported by the Lean mass data that remained constant in both groups over the 6 months of the study. The active group also showed a significant reduction in the amount of abdominal fat at month 3 and 6 compared to placebo (*p* = 0.01 and 0.018). Additionally, visceral, and subcutaneous fat were reduced significantly at month 6 compared to placebo (*p* = 0.018 and 0.006, respectively, Table 2).

### 3.4. Pathology Data

#### 3.4.1. Satiety Hormones

There was no significant difference in Ghrelin, Leptin or Adiponectin between groups at either 3 or 6-months (Table 3).

#### 3.4.2. Cardiometabolic Parameters

There were no differences either within or between groups for any cardiometabolic parameter measured at any time-point.

#### 3.4.3. Safety Data

All safety markers of liver toxicity were within the normal range at baseline and month 3 and 6 for both treatment groups indicating that the standardized extract is well tolerated and has no safety concerns (Table 3).

#### 3.4.4. Fatigue

There was no difference between participant groups for fatigue at any monthly time-point as measured by Visual Analogue Scale.

### 3.5. Dietary Intake and Physical Activity

Participants were given a daily calorie target based on their basal metabolic rate and starting weight. Participants completed a food tracker, and this was checked regularly to ensure compliance. The daily calorie target was adjusted based on weight lost every 2 months. Compliance was similar in each group however based on review of calorie data, participants in both groups did not comply well with the calorie limit with both groups reducing their calorie intake on average by approximately 20–30% of what was required. Both groups in the study reported approximately the same amount of exercise during the 6 months of the trial. The exercise is measured as number of 30-min sessions completed each week. The average number of sessions per week at baseline was 2.0 in the placebo group and 2.3 sessions in the active group, this increased to 3.5 (placebo) and 4.0 (active) sessions at month 3 and remained constant at month 6 (3.3 sessions placebo and 4.0 sessions active group).

### 3.6. Age and Gender Variations

There was no difference in effect between men and women, although it should be noted that there was a higher proportion of women enrolled in the study. To account for differences in pre and post-menopausal women analysis was conducted to determine if there was a difference in effect between women under 50 and over 50. No difference or correlations were seen.

### 3.7. Adverse Event Reporting

Participants completed adverse symptom questionnaires monthly. Five participants in the placebo group reported symptoms including heartburn, diarrhea, constipation, headache and nausea. Among them, two participants withdrew from the study. Two participants in the active group reported symptoms including constipation, nausea, vomiting and headache pain, with one participant withdrawing from the study. 

### 3.8. Sub-Group Analysis

Of the 136 participants enrolled, 102 had a 25 < BMI < 30 kg/m^2^ and were included in a subgroup analysis. All data presented in this section are for those participants with a 25 < BMI < 30 kg/m^2^. Both groups were well matched, with no significant differences between groups for any baseline measures (Table 4).

After 6-months, the active group had a significant reduction in body weight from baseline (4.2%, *p* < 0.05). After 6-months, the placebo group had a non-significant reduction in body weight from baseline (2.3%, *p* = 0.37) (Figure 4A). The active group had a significantly greater body weight loss compared to the placebo group at months 1,2,3,4 and 6 (*p* < 0.026; Table 5, Figure 2). Additionally, 35% of the participants in the active group had a weight loss of more than 5% vs. 24% of the participants in the placebo group.

Both the active and placebo group had a significant reduction in waist (−3.6 cm vs. −2.1 cm, respectively) and hip (−3.2 cm vs. −2.2 cm, respectively) circumference from baseline following 6-month of supplementation (Figure 3A,B, Table 5). However, the active group had a significant reduction in both hip and waist circumference at 6-months compared to the placebo group (Table 5; *p* = 0.033, 0.015, respectively). Compared to baseline values, BMI was significantly reduced in both the active and placebo group after 3 and 6-months of supplementation (*p* < 0.05). At 6-months, the active group had a significantly greater reduction in BMI compared with the placebo group (*p* = 0.041, Figure 4B, Table 5).

Both the active and placebo group had a significant reduction in Fat mass from baseline after 3 and 6-months of supplementation (*p* < 0.05). At 6-months, the active group had a significant reduction in abdominal, visceral and subcutaneous fat mass compared with the placebo group (*p* < 0.05) (Table 5). For the total fat mass there was a between group difference trending toward significance (*p* = 0.06). There were no differences either within or between groups for any pathology or fatigue measures at any time-point and exercise and diet compliance was also the same between groups.

## 4. Discussion

To date, this is the second known study investigating the effects of a standardized extract from “Moro” blood orange (Morosil^®^) on weight reduction in human participants. A previous study supplemented 60 overweight but otherwise healthy adults with either 400 mg of standardized extract or a placebo for 12-weeks [20]. Following 12-week of supplementation, Cardile and colleagues reported significant reductions (*p* < 0.05) in body weight, BMI, waist and hip measurements. The study presented here, aimed to replicate previous results and see if a continuation of the effects could be seen with a longer duration. Subsequently it showed that supplementation over a 6-month period not only produced similar results at the 3-month point, but it continued to provide beneficial effects for the full 6-month trial. Following 6-months of supplementation, both the active and placebo supplemented groups showed significant (*p* < 0.05) improvements in body weight, BMI and waist and hip circumferences. However, after 6-months of supplementation, the active group had a greater weight loss than the placebo. This was supported by the DXA results which showed a significant decrease in fat mass, visceral and abdominal fat at 6-months compared to placebo. These results translated to a significant reduction in BMI for both groups at both month 3 and month 6, with the active group having a significantly lower BMI than the placebo group at month 6. A significant reduction in BMI in the active group was also shown by Cardile after 4 weeks, however in that study no significant reduction was seen at any time points in the placebo group [20]. Weight loss findings were further reflected in the waist and hip circumferences, these were also consistent with the findings by Cardile and colleagues [20]. In the earlier study, participants in both the active and placebo group had significant reductions in hip and waist measurements at 12 weeks (*p* < 0.05). Following 6-months of supplementation in this trial, both groups had a significant reduction in waist and hip circumference. However, the active group had a greater reduction in both waist (*p* < 0.05) and hip (*p* < 0.05) circumference at 6-months compared to the placebo. Sub-group analysis of participants in the overweight range only (25 < BMI < 30 kg/m^2^) were similar to the total group analysis indicating the treatment works consistently in a BMI range of 25–33 kg/m^2^. The above-reported results confirmed the experimental data present in the literature on the role of the bioactive compounds present in Moro blood orange extract in promoting weight management and preventing obesity through the regulation of both lipolytic and lipogenic genes [29]. The Phytocomplex present in Moro orange mainly composed of flavonoids such as anthocyanins, naringenin and hesperetin, is able in adipocytes to reduce lipids accumulation and to modulate adipocytokines release with a concomitant reduction of inflammation and oxidative stress [30]. Particularly, Cyanidin-3-glucoside along with the aforementioned molecular effects was demonstrated to improve also mitochondrial biogenesis, which is strictly involved in the modulation of lipid metabolism [31]. The synergistic action exerted by the phytocomplex results in a significant reduction in adipocytes size and a restoration of cellular homeostasis [30]. All safety markers of liver toxicity were within the normal range at baseline and month 3 and 6 for both treatment groups indicating that the Moro orange standardized extract is well tolerated and has no safety concerns. Gastrointestinal symptoms were reported in a small number of trial participants (placebo *n* = 5, active *n* = 1). These symptoms were not reported in the 2015 trial [20] and the incidence is significantly below the current prevalence of gastrointestinal symptoms in the healthy population [32]. The participant selection and grouping criteria used in this trial suggest that the benefits of 400 mg orally daily of the standardized extract will be experienced by the general ‘healthy’ adult population. Participants were given a daily calorie target based on their Basal metabolic rate and starting weight. Participants also completed a food tracker, and this was checked regularly to ensure compliance. The daily calorie target was adjusted based on weight lost every 2 months. However, both groups did not firmly adhere to the recommended diet and exercise regime during the 6-month trial. This result however may not be too surprising. While the participants recruited were interested in losing weight, they may not have been psychologically ready to undertake such lifestyle changes. As such, the observed compliance to the prescribed diet and exercise is likely the start of a lifestyle change that may need to be made at a slower rate than introduced in this trial. Despite this, while the reduced compliance may have affected the amount of weight the participants could potentially have lost, as each group had an equivalence adherence to the prescribed protocol, it can still be assumed that the additional benefits seen in the active group is due to the supplementation and not an external factor confirming the effectiveness of Moro blood orange extract when is associated with regular physical activity and a healthy balanced diet. As with all studies, there were limitations with this study. First, there was a 30% withdrawal rate of participants in the first 3 months of the study. This could be attributed to the amount of baseline assessment data that was required to be collected and participants not wanting to adhere to dietary and lifestyle advice. Second, participant exercise and diet data were self-reported. Third, comparative to other weight loss trials, 6-months was a relatively short period. Some studies range from 12 to 18 months. Last, there were limited Moro blood orange studies on weight reduction in human participants for comparison of our findings.

## 5. Conclusions

In the present study, “Moro” blood orange standardized extract, in conjunction with diet and exercise, was shown to be safe and well tolerated. The secondary metabolites present in the active ingredient by regulating lipid metabolism through fatty acid biosynthesis and oxidation promoted increased weight loss and reduced waist and hip circumference in overweight but otherwise healthy male and female participants compared to placebo. As reported by the scientific literature, this effect is probably linked with the activity and modulation at the gene level exerted by the phytocomplex in a combined way. Lastly, further pre-clinical and clinical studies could be useful to prove the efficacy of “Moro” blood orange standardized extract (Morosil^®^) supplementation in weight management.

In conclusion, results confirmed that the supplementation with the standardized extract may significantly contribute as a complementary strategy in weight management programs.

## Figures and Tables

**Table 1:** Baseline participant details.

	Active (*n* = 65)	Placebo (*n* = 71)
Female (*n*)	50	49
Male (*n*)	15	22
Age (years)	52.9 ± 8.4	51.9 ± 10.2
Asian Heritage (*n*) ^	8	8
Systolic Blood pressure (mmHg)	126 ± 16.2	128 ± 13.8
Diastolic Blood pressure (mmHg)	84.8 ± 10.3	83.8 ± 9.3
Resting heart rate (BPM)	69.9 ± 9	67.6 ± 9
BMI (Range)	25–32.9 m/h^2^	25–33.1 m/h^2^

Values represented as mean ± SD; BPM = beats per minute. ^ All other participants Caucasian.

**Table 2:** Anthropometry data for all trial participants.

	Active			Placebo		
	Baseline	Month 3	Month 6	Baseline	Month 3	Month 6
WC (cm)	101.1 ± 9.9	98.5 ± 10.5 ^#^	97.2 ± 10.7 *^#^	103.4 ± 11.0	101.3 ± 11.4 ^#^	101.7 ± 11.7 ^#^
HC (cm)	113.4 ± 6.9	111.0 ± 7.5 ^#^	110.1 ± 7.5 *^#^	115.6 ± 7.2	113.6 ± 6.7 ^#^	113.5 ± 7.2 ^#^
Weight (kg)	88.4 ± 11.2	85.5 ± 11.5 ^#^	84.7 ± 11.7 *^#^	90.8 ± 13.8	89.0 ± 14.1 ^#^	88.8 ± 14.5 ^#^
Weight loss (%)	−3.2 ± 3.7 ^#^	−4.2 ± 5.0 *^#^		−2.1 ± 3.3	−2.2 ± 4.2 ^#^	
BMI (kg/m^2^)	29.5 ± 1.6	28.6 ± 2.0 ^#^	28.3 ± 2.2 *^#^	29.4 ± 1.4	28.9 ± 1.7 ^#^	28.8 ± 1.9 ^#^
Lean Mass (kg)	54.0 ± 11.3	52.5 ± 10.1	52.4 ± 10.2 ^#^	56.1 ± 13.0	55.4 ± 12.8	55.6 ± 13.2 ^#^
FM (kg)	32.1 ± 7.2	30.6 ± 6.4 *^#^	29.7 ± 6.7 *^#^	33.1 ± 7.5	31.8 ± 8.0 ^#^	31.2 ± 8.2 ^#^
FM Arm (kg)	3.9 ± 1.1	3.7 ± 0.9	3.6 ± 0.9 ^#^	3.7 ± 1.0	3.6 ± 1.0	3.5 ± 1.0 ^#^
FM Leg (kg)	11.9 ± 3.3	11.4 ± 3.2	11.4 ± 3.8 ^#^	12.2 ± 3.8	11.5 ± 4.0	11.3 ± 3.6 ^#^
FM abdominal (trunk) (kg)	15.9 ± 3.3	14.6 ± 3.5 *^#^	14.1 ± 3.5 *^#^	16.3 ± 4.1	15.6 ± 4.4 ^#^	15.3 ± 4.4 ^#^
Android Fat (kg)	3.6 ± 1.8	2.7 ± 1.0 ^#^	2.6 ± 1.0 ^#^	4.0 ± 2.1	3.1 ± 1.4	3.0 ± 1.4 ^#^
Gynoid Fat (kg)	7.0 ± 3.8	5.2 ± 1.5 ^#^	5.1 ± 1.5 ^#^	7.7 ± 4.0	5.9 ± 2.8 ^#^	5.9 ± 2.8 ^#^
Visceral Fat (g)	632.9 ± 223.5	588.6 ± 245.3 ^#^	554.2 ± 232.4 *^#^	632.8 ± 249.6	581.5 ± 256.6 ^#^	575.8 ± 254.9 ^#^
Visceral Fat Area (cm^2^)	131.4 ± 46.4	122.0 ± 51.0	115.6 ± 47.4 ^#^	131.5 ± 52.0	120.1 ± 53.1	118.6 ± 52.6 ^#^
Subcutaneous Fat (kg)	15.2 ± 3.1	14.0 ± 3.4 ^#^	13.5 ± 3.4 *^#^	15.7 ± 3.9	15.0 ± 4.2 ^#^	14.7 ± 4.3 ^#^

^#^ Indicated significant decrease from baseline within group (*p* < 0.05); * indicates significant difference between groups (*p* < 0.05) in ITT analysis; (DEXA measures baseline placebo *n* = 69, active *n* = 62; 3- and 6-month placebo *n* = 56, active *n* = 46). WC = waist circumference; HC = hip circumference; BMI = body mass index; FM = fat mass.

**Table 3:** Pathology results for the active and placebo group for all participants.

	Active			Placebo		
	Baseline	Month 3	Month 6	Baseline	Month 3	Month 6
ALT (U/L)	25.9 ± 15.6	20.9 ± 7.9	23.3 ± 9.1	25.9 ± 11.5	23.6 ± 10.6	24.7 ± 10.0
AST (U/L)	23.7 ± 7.2	23.4 ± 8.2	25.8 ± 9.1	22.2 ± 9.8	22.3 ± 8.9	23.1 ± 10.4
GGT (U/L)	29.7 ± 15.3	26.3 ± 14.7	24.3 ± 13.9	36.4 ± 25.7	33.1 ± 28.2	32.7 ± 28.3
TBIL (umol/L)	11.9 ± 4.5	11.9 ± 4.7	12.3 ± 5.6	11.7 ± 5.0	11.1 ± 4.8	11.0 ± 3.9
Cholesterol (umol/L)	5.8 ± 1.1	5.7 ± 1.1	5.7 ± 1.2	5.8 ± 1.2	5.6 ± 1.1	5.7 ± 1.1
HDL (mmol/L)	1.8 ± 0.4	1.8 ± 0.5	1.9 ± 0.6	1.7 ± 0.5	1.6 ± 0.5	1.7 ± 0.4
LDL (pg/mL)	3.6 ± 0.9	3.5 ± 0.9	3.5 ± 0.9	3.7 ± 1.0	3.5 ± 1.0	3.5 ± 0.8
TRI (mmol/L)	1.0 ± 0.4	1.0 ± 0.5	1.1 ± 0.7	1.4 ± 0.7	1.3 ± 0.7	1.3 ± 0.7
Glucose (mmol/L)	5.6 ± 1.0	5.6 ± 0.8	5.5 ± 0.9	5.6 ± 1.0	5.6 ± 0.8	5.7 ± 0.8
Insulin (mU/L)	12.2 ± 8.1	13.0 ± 9.9	11.4 ± 7.9	11.2 ± 6.6	12.0 ± 7.8	12.7 ± 8.4
Creatine (umol/L)	92.4 ± 11.8	96.4 ± 21.8	94.0 ± 13.3	90.9 ± 18.1	94.2 ± 15.4	93.2 ± 14.6
Ghrelin (ng/mL)	0.41 ± 0.32	0.47 ± 0.51	0.49 ± 0.47	0.40 ± 0.36	0.39 ± 0.34	0.40 ± 0.38
Leptin (ng/mL)	5.71 ± 5.13	5.10 ± 5.72	5.51 ±7.11	4.77 ±4.10	4.06 ± 3.50	3.9 ±3.64
Adiponectin (ug/mL)	6.26 ± 3.77	6.43 ± 4.11	6.35 ± 3.62	5.22 ± 3.59	5.21 ± 3.50	5.54 ± 3.83

ALT = Alanine transaminase, AST = Aspartate aminotransferase, GGT = Gamma-glutamyl transferase, TBIL = total bilirubin, GLU = glucose, TRI = triglycerides, HDL = high density lipoproteins, LDL = low density lipoproteins. Values represented as mean ± SD.

**Table 4:** Baseline participant details for participants with a 25 < BMI < 30 kg/m^2^.

	Active (*n* = 48)	Placebo (*n* = 54)
Female (*n*)	41	40
Male (*n*)	7	14
Age (years)	53.1 ± 7.5	52.0 ± 10.1
Asian Heritage (*n*)	7	8
Systolic Blood pressure (mmHg)	126 ± 16.2	124.2 ± 16.4
Diastolic Blood pressure (mmHg)	84.8 ± 10.3	83.8 ± 9.8
Resting heart rate (BPM)	69.9 ± 9	69.9 ± 9.3

Values represented as mean ± SD; BPM = beats per minute.

**Table 5:** Anthropometry Data for Participants with a BMI > 25 – < 30 kg/m^2^.

	Active			Placebo		
	Baseline	Month 3	Month 6	Baseline	Month 3	Month 6
WC (cm)	98.6 ± 9.0	96.1± 9.7 ^#^	95.0 ± 10.2 *^#†^	101.8 ± 11.1	99.4 ± 11.1 ^#^	99.8 ± 11.2^#^
HC (cm)	112.8 ± 7.2	110.5 ± 8.0 ^#^	109.7 ± 8.1 *^#†^	114.7 ± 7.3	112.6 ± 6.8^#^	112.5 ± 7.05 ^#^
Weight (kg)	85.5 ± 10.3	82.7 ± 10.5 *^#†^	81.9 ± 10.9 *^#†^	87.8 ± 13.4	86.1 ± 14.0 ^#^	85.83 ± 14.02 ^#^
Weight loss (%)		−3.3 ± 3.7 ^#†^	−4.2 ± 5.2 ^#^*^†^		−2.1 ± 3.4	−2.3 ± 4.0 ^#^
BMI (kg/m^2^)	28.8 ± 1.4	28.0 ± 1.8 ^#†^	27.7 ± 2.1 *^#†^	28.9 ± 1.2	28.4 ± 1.6 ^#^	28.3 ± 1.7 ^#^
Lean Mass (kg)	51.0 ± 9.1	50.0 ± 8.0	50.1 ± 8.3	52.9 ± 11.6	52.7 ± 11.6	52.8 ± 11.7
FM (kg)	32.5 ± 6.3	30.6 + 6.7 ^#^	29.9 ± 7.0 ^#^	33.2 ± 8.0	31.5 ± 8.3 ^#^	31.0 ± 8.5 ^#^
FM Arm (kg)	3.9 ± 1.2	3.7 ± 0.9	3.7 ± 1.0	3.8 ± 1.0	3.6 ± 1.0	3.6 ± 1.0
FM Leg (kg)	12.2 ± 3.2	11.7 ± 3.1	11.8 ± 3.9 ^#^	12.4 ± 3.9	11.4 ± 4.0	11.4 ± 3.7 ^#^
FM abdominal (trunk) (kg)	15.6 ± 3.5	14.4 ± 3.8 ^#^	14.0 ± 3.8 *^#†^	16.2 ± 4.5	15.3 ± 4.6 ^#^	15.1 ± 4.6 ^#^
Android Fat (kg)	3.6 ± 2.0	2.6 ± 1.1 ^#^	2.5 ± 1.0 ^#^	3.8 ± 2.0	3.1 ± 1.5	3.0 ± 1.5 ^#^
Gynoid Fat (kg)	7.3 ± 3.8	5.4 ± 1.5 ^#^	5.3 ± 1.6 ^#^	7.5 ± 3.7	6.1 ± 3.0 ^#^	6.1 ± 3.0 ^#^
Visceral Fat (g)	662.2 ± 207.6	641.6 ± 234.8 ^#^	596.0 ± 229.8 *^#†^	686.3 ± 251.2	637.1 ± 261.4	628.9 ± 260.1 ^#^
Visceral Fat Area (cm^2^)	125.1 ± 41.6	116.3 ± 49.4	107.6 ± 43.6	124.7 ± 51.0	112.4 ± 47.9	111.4 ± 47.1
Subcutaneous Fat (kg)	14.9 ± 3.4	13.8 ± 3.6 ^#†^	13.5 ± 3.6 *^#†^	15.6 ± 4.3	14.8 ± 4.5	14.6 ± 4.5 ^#^

^#^ Indicates significant decrease from baseline (*p* < 0.05); * indicates significant difference in between groups in ITT analysis (*p* < 0.05); ^†^ indicates significant difference between groups in PP analysis (*p* < 0.05); WC = waist circumference; HC = hip circumference; BMI = body mass index; FM = fat mass.

## Data Availability

The data presented in this study is available on request.

## Supplementary Materials

The following are available online at https://www.mdpi.com/article/10.3390/nu14030427/s1, Certificate of authentication, detailed product specification and some parameters on purity.
