# Supplementary material for: Effectiveness of “Moro” Blood Orange Citrus sinensis Osbeck (Rutaceae) Standardized Extract on Weight Loss in Overweight but Otherwise Healthy Men and Women—A Randomized Double-Blind Placebo-Controlled Study
Source: Nutrients. 2022 Jan 18;14(3):427. doi: 10.3390/nu14030427 (PMC8838101; doi:10.3390/nu14030427)
Supplement: Supplementary file 1 [file nutrients-14-00427-s001.zip › nutrients-1551561-supplementary.pdf]

Certificate of Authentication

Certificate of Authentication Number (COA): P001.1-BIO00082020

Client: **Bionap Srl**
Audit date: 09-29-2020
Auditor(s): **Dr. Steven Newmaster (Chief Science Officer, TRU-ID Ltd.)**
Report generated: 11-25-2020
Lot: 08202008-10

Molecular Diagnostic Test:
DNA concentration will be assessed using a Qubit Fluorometer in addition to the TRU-ID DNA authentication test using TRU-ID mini-sequence analysis technology. Molecular diagnostics in this test used Sanger sequencing of standardized DNA sequences for plants. Samples were analyzed against the TRU-ID Plant DNA sequence library.

Authenticated:
The submitted sample name, and its molecular patterns agree with molecular patterns and sample names of TRU-ID reference material and Plant DNA sequence library.

TEST RESULTS:

Certificate of Authentication Numbers (COA): P001.1-BIO00082020

Product Name: Morosil

Latin Binomial: *Citrus sinensis*

Molecular Diagnostic Test Result: Positive

TRU-ID Certification Standard: Verified Compliant

The results and conclusions presented in this report are true and accurate to the best of TRU-ID's knowledge. However, the advanced molecular diagnostics employed can very rarely give rise to inconclusive, or incongruent results, and in such cases corroboration of this result by repeat testing, or different analytical methods may be warranted. This report should not be copied or reproduced, except in its entirety.

PHYSICAL-CHEMICAL PROPERTIES	
PARAMETERS	STANDARD VALUES
Form	Powder
Color	Red purple
Taste	Acid
Smell	Characteristic
Moisture	≤ 5%
Ashes	≤ 1.2%
Heavy metals:	Conform to Reg. EC 1881/2006 and subsequent modifications and supplements
Lead (Pb)	< 3 ppm
Arsenic (As)	< 3 ppm
Cadmium (Cd)	< 1 ppm
Mercury (Hg)	< 0.1 ppm
Pesticides	Conform to Reg. EC 396/2005 and subsequent modifications and supplements
PAH	Conform to Reg. EC 1881/2006 and subsequent modifications and supplements
Benzo(a)pyrene	< 10 µg/kg
Sum of Benzo(a)pyrene, Benz(a)anthracene, Benzo(b)fluoranthene and chrysene	< 50 µg/kg
pH (2,5 % aqueous solution)	2.1 – 3.2
Solubility	Moderately soluble in water
Residual solvents (ethanol)	Less than 0.2%
Particle size	More than 90% pass 300 micron
Radioactivity	The product is not irradiated

MICROBIOLOGICAL SPECIFICATIONS	
PARAMETERS	STANDARD VALUES
Total plate count	< 1000 cfu/g
Yeasts and Moulds	< 100 cfu/g
Enterobacteriaceae	< 100 cfu/g
Escherichia coli	Absent
Pseudomonas aeruginosa	Absent
Staphylococcus aureus	Absent
Salmonella specie	Absent

TOXICOLOGICAL EVALUATION	
LD_{50} (in rats)	> 2 g/Kg
Primary skin irritation	No irritant<br>Occlusive Patch method (25 volunteers)
Cytotoxicity	No toxic<br>Epithelial human cells (Caco-2)

ADDITIONAL DATA

GMO free: according to Reg. UE 1829/2003 and Reg. UE 1830/2003.
